# Supplementary material for: Were domestic camelids present on the prehispanic South American agricultural frontier? An ancient DNA study
Source: PLoS One. 2020 Nov 5;15(11):e0240474. doi: 10.1371/journal.pone.0240474 (PMC7644007; doi:10.1371/journal.pone.0240474)
Supplement: Attachment — Submitted filename: Response to Reviewers.pdf [file pone.0240474.s003.pdf]

*Review of "***Were domestic camelids present on the prehispanic South American agricultural frontier? An ancient DNA study***"* (PONE-D-20-06090)

Dear David Caramelli
Academic Editor
PlosOne

Thank you for the review process. We appreciate your effort and the reviewer work in order to improve our manuscript. We had considered all the reviewers point (as we detail below). We think that these recommendations make a better manuscript.

JOURNAL REQUIREMENTS

1. *Please ensure that your manuscript meets PLOS ONE's style requirements, including those for file naming.*

It has been revised

2. *In your manuscript, please provide additional information regarding the specimens used in your study. Ensure that you have reported specimen numbers and complete repository information, including museum name and geographic location.*

The code museum is the same that we have used. Geographic location has been described on Fig 2 and S2 Table.
'No permits were required for the described study, which complied with all relevant regulations.' Was added (Line 184, under Table 1)

3. *We note that you have stated that you will provide repository information for your data at acceptance. Should your manuscript be accepted for publication, we will hold it until you provide the relevant accession numbers or DOIs necessary to access your data. If you wish to make changes to your Data Availability statement, please describe these changes in your cover letter and we will update your Data Availability statement to reflect the information you provide.*

The raw data are in the SRA accession PRJNA603673. The link is https://dataview.ncbi.nlm.nih.gov/object/PRJNA603673?reviewer=r0stc5g54tm9ttaj0cp96n4klj

4. *Thank you for stating the following in the Acknowledgments Section of your manuscript:*

It was corrected

5. *We note that Figures 1 and 2 in your submission contain map/satellite images which may be copyrighted.*

Maps were built on QGIS software based on NASA open data (https://earthdata.nasa.gov).

REVIEWER: 1

I have to signal only a couple of typos: line 90 "presence" and S1 Table "Provenience"
The line 90 "presence" and S1 Table with "Provenience" was corrected the typos

I would like to see in S1 Table at least the median length of the sequenced molecules mapping on the target.
We have added in S2 Table (samples sequenced in this study, S1 Table belong to sequences used from GenBank) a column with number fragments mapped to reference, total length of mapped fragments and average fragment length

I also suggest to spend some words to briefly describe the DNA extraction method, since it is not clear only through the cited reference.
The reviewer must have missed the information in the original submission. We describe the DNA extraction method in detail from lines 190 to 217.

It is a pity that the authors could not analyse any sample from Site LD-S4 that they mentioned as the one with evidence of domestic camelids according to osteometric evidence. Were those samples not available for the analysis?
We agree with the reviewer; we could not obtain the permission.

REVIEWER: 2

1) In the abstract it would be better to indicate the number of samples for which the authors obtained results (41?) and not the total samples analysed (52?), or at least to specify that.
It was corrected

2) In Table 1 I think that the authors should:
(i) add a colum where specify what kind of skeletal element was analysed (e.g. petrous bone, long bone, tooth...); **It was added**

(ii) add a column where they indicate if the chronology is based on radiocarbon dating or material culture; **It was described below Table 1**
(iii) check the number of samples, because in the abstract and results the total number of samples is 53 and the ones with sufficient endogenous is 41, but in the table 1 there are 53 samples, of which 10 with sufficient endogenous;
(iv) explain why some samples have the same ID, are they referred to the same individual?
(iii and iv) 19, 78, 31 were done duplicated for NGS.
41 was used for phylogenetic analyses, using the sequence available for two loci, specifically cyt*b* (1,140 bp) and D-loop (1,215 bp)

3) The authors followed proper ancient DNA guidelines, but I suggest to add the results from MapDamage software to analyzse damage patterns with the aim to authenticate the data here obtained.

While we agree with the reviewer that being able to visualize damage patterns along mapped reads using a program such as MapDamage is helpful, we are unable to employ due to the method used for mapping. We used the mapping iterative assembler (MIA, https://github.com/mpieva/mapping-iterative-assembler) for mapping merged reads to the mtDNA reference genome, which does not generate the necessary file type required for running MapDamage (SAM/BAM format). MIA was originally developed for working specifically with ancient DNA (i.e., Neanderthal mtDNA) and provides a number of additional steps that help minimize erroneous SNP calls when working with ancient DNA. For example, the method uses a position specific substitution matrix that has been altered to work with damaged/ancient DNA to help reduce potential calling errors. The method will iterate until convergence on a consensus sequence, which is one of the main selling points of this method when mapping ancient mtDNA.

To further reduce potential erroneous SNP calls associated with cytosine deamination, "total DNA was treated with 1X USER enzyme mix (New England BioLabs) for 3h at 37°C to reduce nucleotide substitution errors …., which is common when working with aDNA [74,75]" (line 211). This mix combines two enzymes, the Uracil-DNA glycosylase and DNA glycosylase-lyase endonuclease VIII and replaces with abasic sites the cytosines that have been deaminated post-mortem into uracils. So, the transitions C-T has been corrected, which is what MapDamage is used to show when not corrected.

4) Given that the identification of domestic from wild species through DNA analysis is often complicated and confused because of recent times of divergence and/or hybridisation phenomena, the paper will benefit from some more sentences about the power of mitochondrial DNA analysis in the identification of Lama glama and Lama guanicoe. For example, the authors stated that "previous research suggests that it is less likely to occur in the wild because haplotypes are uncommon between guanacos and vicuñas", but are there shared or private mitochondrial haplotypes between Lama glama and Lama guanicoe?

There are sufficient differences between llama and guanaco at the cyt*b* and D-loop to result in reciprocal monophyly in the gene trees as shown on Fig 3. In contrast to previous studies, we have used both markers, the cyt*b* and D-loop, together increasing the conserved sites and maintaining the polymorphisms.

"As shown previously using mtDNA data [84], guanaco form a monophyletic clade separate from vicuña and domestic llama..." (line 293)

5) add more keywords, for example the name of the species, domestication, mitochondrial DNA, the geographic area, etc,

The suggestions have been done

6) in the caption of the Figure 3 add an explanation about the use of the colors blue, red, green and black for the samples'ID.
"Sample names are color coded according to whether the ancient sample was sequenced for this study (blue) and originated near the high altitude LD-S4 site (red), or were contemporary (black) or ancient (green) mtDNA sequences obtained from GenBank." (Fig 3)

Minor edits
The paper will benefit from a revision of the English, I suggested here some words or sentences that need to be revised.
- the term "presense" need to be changed in "presence" (e.g. lines 59, 67, 90, 92, etc)
- line 61 " Traditionally, In South..." change in "Traditionally, in South...
- check "reducancy" at line 245 **the word is "reduced"**
- check the concentration of the library (9 ng/ml) line 211
- line 252 check the sentence "41 sampled ancient Holocene samples sequenced" and specify that 41 are referred to the samples analysed in the paper.
- check "socieites" at line 351
- line 389 check "intead"

All of the above indicated edits have been implemented in the revised manuscript.

These changes have clearly improved our manuscript. Thank you again for consideration of our revised manuscript.

Best regards,

Cinthia C. Abbona, PhD
Archaeogenetic Department
IDEVEA (CONICET-UTN)
Av. Urquiza 314
CP M5602GCH
San Rafael, Mendoza Argentina
e-mail abbonacinthia@gmail.com
